# Orbital Trapdoor Fracture: An Open-and-shut Case?

**DOI:** 10.5811/cpcem.2016.11.32397

**Published:** 2017-01-17

**Authors:** Shane M. Summers, Richard M. Wood, Justin E. Costello, Christian L. Carlson

**Affiliations:** San Antonio Military Medical Center, Department of Emergency Medicine, JBSA Fort Sam Houston, Texas

## INTRODUCTION

A trapdoor fracture is a rare condition defined as a minimally displaced fracture of the orbital floor that has spontaneously reduced to its original position incarcerating an extraocular muscle.^1^ Clinicians have described the trapdoor fracture as a “white-eyed blowout” because of a paucity of physical examination abnormalities.^2^ Further complicating diagnosis, the injury may be radiographically occult on orbital computed tomography (CT).^3^ Trapdoor fractures are important for the emergency physician to identify because urgent surgical repair is recommended to reduce morbidity.

## CASE REPORT

**Image 1:**
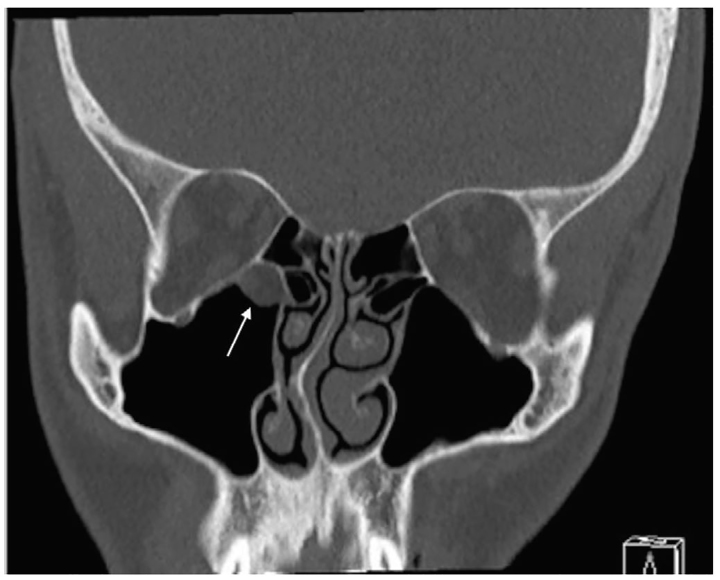
Coronal non-enhanced computed tomography in bone window: Soft tissue density noted in the maxillary antrum and no definite fracture. Lesion initially called a mucosal retention cyst (arrow).

**Image 2:**
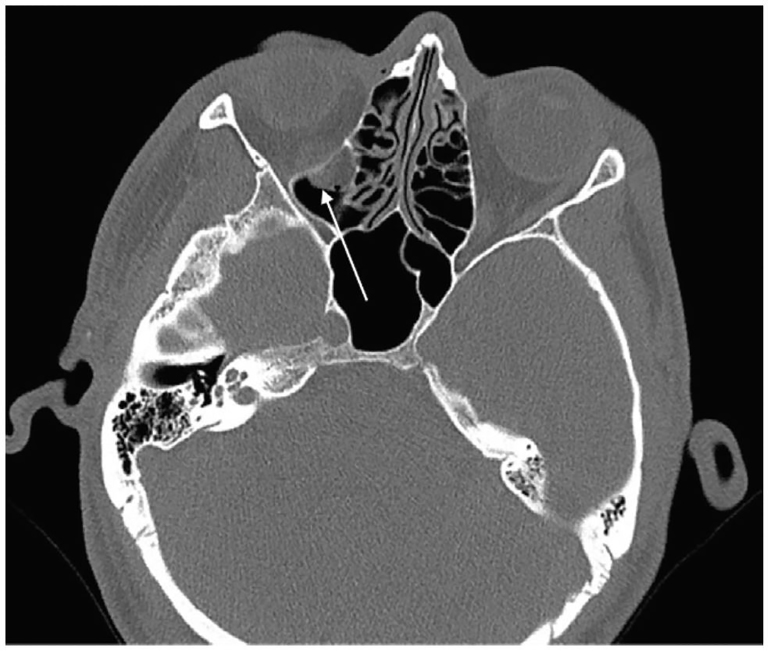
Axial non-enhanced CT in bone window: Soft tissue density again noted in the maxillary antrum (arrow). No definite fracture.

A 26-year-old man presented with binocular vertical diplopia after an assault. He denied loss of consciousness, headache, or vomiting. Physical examination revealed a Glasgow Coma Scale of 15, normal visual acuity and pupils, mild periorbital ecchymosis, and restricted right ocular motion in temporal downward gaze. There was no injection of the sclera, bony step-off, enophthalmos, or proptosis. Head and orbital CT were reported as negative for injury. A maxillary mucosal retention cyst was incidentally noted on CT (Images 1 and 2).

**Image 3:**
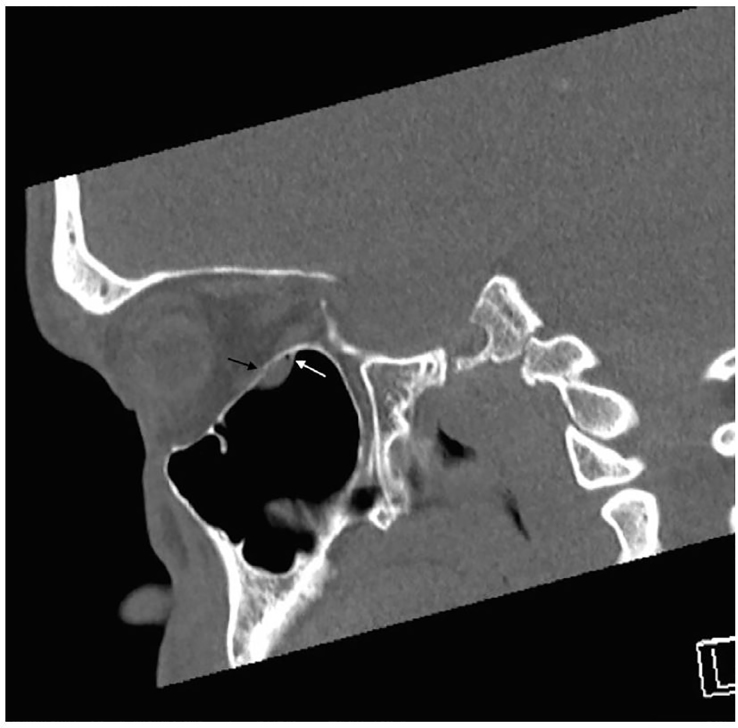
Sagittal non-enhanced CT in bone window: Subtle defect in orbital floor (black arrow) with small focus of air adjacent to soft tissue density (white arrow).

**Image 4:**
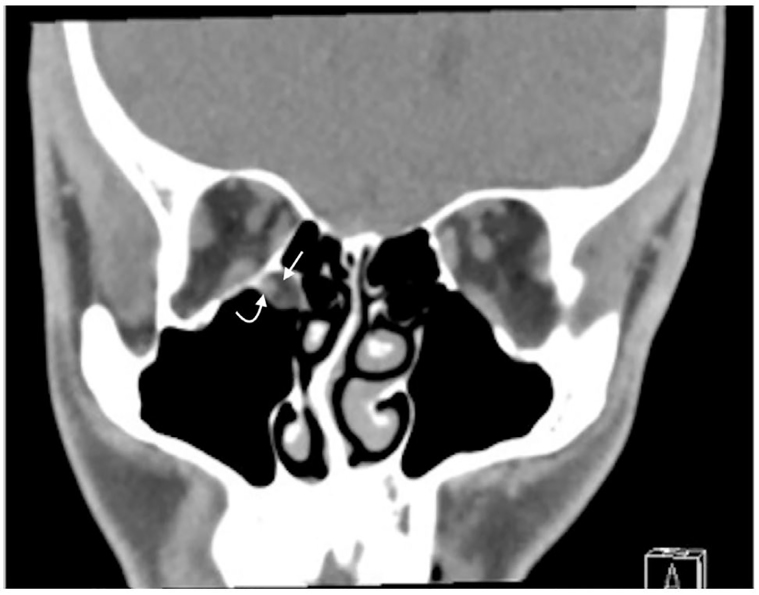
Coronal non-enhanced CT in soft tissue window: Inferior rectus muscle (curved arrow) and orbital fat (straight arrow) in the extra-orbital maxillary antrum.

Ophthalmology was consulted and performed forced duction testing, which confirmed gaze restriction. The patient was taken to the operating room where a trapdoor fracture with entrapment of the inferior rectus muscle was diagnosed and surgically repaired. Retrospective review of the CT revealed that the soft tissue density misdiagnosed as a maxillary sinus cyst displayed radiodensity characteristics consistent with herniation of orbital fat and entrapped muscle fibers (Images 3 and 4).

## DISCUSSION

Because of their relative bony elasticity, trapdoor fractures are reported much more frequently in children, whereas adults tend to present with readily apparent comminuted fracture patterns.^4^ However, more recent literature suggests this injury is not exclusive to pediatric patients and can occur in young adults.^5^–^7^ Trapdoor fractures may be missed unless extraocular muscles are tested through the full range of motion and close attention is paid to the location of the orbital soft tissues on CT.^3^ The emergency physician must consider this diagnosis in younger patients with orbital trauma and abnormal ocular motility, even with a non-diagnostic CT, because operative intervention within 24 hours is associated with improved outcomes.^8^ Reported complications of delayed surgical repair include residual gaze restriction and diplopia secondary to ischemia of the extraocular muscle.^9^
